# Decreased Plasma Hydrogen Sulfide Level Is Associated With the Severity of Depression in Patients With Depressive Disorder

**DOI:** 10.3389/fpsyt.2021.765664

**Published:** 2021-11-11

**Authors:** Yuan-Jian Yang, Chun-Nuan Chen, Jin-Qiong Zhan, Qiao-Sheng Liu, Yun Liu, Shu-Zhen Jiang, Bo Wei

**Affiliations:** ^1^Biological Psychiatry Laboratory, Jiangxi Mental Hospital/Affiliated Mental Hospital of Nanchang University, Nanchang, China; ^2^Department of Psychiatry, Jiangxi Mental Hospital/Affiliated Mental Hospital of Nanchang University, Nanchang, China; ^3^Jiangxi Provincial Clinical Research Center on Mental Disorders, Nanchang, China; ^4^Department of Neurology, The Second Clinical Medical College, The Second Affiliated Hospital, Fujian Medical University, Quanzhou, China

**Keywords:** depression, hydrogen sulfide (H_2_S), plasma, severity, correlation

## Abstract

Accumulating evidence has suggested a dysfunction of synaptic plasticity in the pathophysiology of depression. Hydrogen sulfide (H_2_S), an endogenous gasotransmitter that regulates synaptic plasticity, has been demonstrated to contribute to depressive-like behaviors in rodents. The current study investigated the relationship between plasma H_2_S levels and the depressive symptoms in patients with depression. Forty-seven depressed patients and 51 healthy individuals were recruited in this study. The 17-item Hamilton Depression Rating Scale (HAMD-17) was used to evaluate depressive symptoms for all subjects and the reversed-phase high-performance liquid chromatography (RP-HPLC) was used to measure plasmaH_2_S levels. We found that plasma H_2_S levels were significantly lower in patients with depression relative to healthy individuals (*P* < 0.001). Compared with healthy controls (1.02 ± 0.34 μmol/L), the plasma H_2_S level significantly decreased in patients with mild depression (0.84 ± 0.28 μmol/L), with moderate depression (0.62 ± 0.21μmol/L), and with severe depression (0.38 ± 0.18 μmol/L). Correlation analysis revealed that plasma H_2_S levels were significantly negatively correlated with the HAMD-17 scores in patients (*r* = −0.484, *P* = 0.001). Multivariate linear regression analysis showed that plasma H_2_S was an independent contributor to the HAMD-17 score in patients (*B* = −0.360, *t* = −2.550, *P* = 0.015). Collectively, these results suggest that decreased H_2_S is involved in the pathophysiology of depression, and plasma H_2_S might be a potential indicator for depression severity.

## Introduction

Depression is a common illness with more than 264 million people affected in the worldwide (1). Person with depressive disorder experiences depressed mood, loss of interest and enjoyment, and reduced energy leading to diminished activity for at least 2 weeks. Depression results from a complex interaction of social, psychological and biological factors (2). Although the neurobiological mechanisms underlying depression have not been recognized completely, emerging evidence suggests a dysfunction of synaptic plasticity in the pathophysiology of depression (3–5). For example, exposure to chronic stress was shown to induce dendritic atrophy and spine loss in the hippocampus and prefrontal cortex (6–8). Impaired long-term potentiation (LTP) was observed in the hippocampus of the chronic stress mice model of depression (9). Restoration of stress-induced changes in synaptic plasticity within the corticoaccumbal glutamate circuit prevented the behavioral vulnerability of mice to chronic stress (10).

Synaptic plasticity is an experience-dependent change in synaptic strength at preexisting synapses, in which one type of ionotropic glutamate receptors, N-methyl-D-aspartate receptor (NMDAR), plays a key role (11). Numerous studies have reported that there are abnormal gene expression and function in NMDARs in the hippocampus of depressed patients (12–14). Chronic stress could reduce the expression of NMDARs in the hippocampus in rodents (15–17). Preclinical studies indicate that both acute and chronic stress can perturb the normal balance between synaptic potentiation and depression in hippocampal pyramidal neurons (18–20). Furthermore, a number of experimental and clinical studies have demonstrated that improving actions of antidepressants are associated with restoration of maladaptive brain plasticity (21–23).

Hydrogen sulfide (H_2_S) is a member of the gasotransmitter family that is associated with the maintenance of neuronal plasticity, excitability, and homeostatic functions (24). It is mainly produced by the enzyme cystathionine-β-synthase (CBS) in the brain and the enzyme cystathionine-γ-lyase (CSE) in the peripheral tissues (25). Abe and Kimura first demonstrated the influences of H_2_S on synaptic plasticity. They showed that physiological concentrations of H_2_S facilitated the induction of hippocampal LTP by increasing the activity of NMDARs (26). Inhibition of H_2_S generation would lead to a reduction in NMDAR-mediated synaptic response and cause an impairment of LTP in the amygdala (27). Gas can freely diffuse across cell membranes and blood-brain barrier. Previous studies have demonstrated that intraperitoneal injection of NaHS (an H_2_S donor) or inhalation of H_2_S can increase brain H_2_S content and promote amygdalar LTP and emotional memory in rats (28), and systemic administration of NaHS could elevate hippocampal H_2_S level and dramatically reversed the cognitive and synaptic plasticity deficits in APP/PS1 transgenic mice (29).

Since H_2_S has an important regulatory role in synaptic plasticity, some studies have explored its role in depression. Chen et al. reported that chronic intraperitoneal treatment with NaHS produced a specific antidepressant-like effect in non-stressed mice and rats (30). Administration of NaHS significantly alleviated the depressive-like behaviors in streptozotocin-induced diabetic rats (31). Moreover, a recent study showed that decreased level of endogenous H_2_S in the hippocampus was responsible for the abnormal behaviors induced by chronic unpredictable mild stress, and the depressive-like behavior of rats could be alleviated within a few hours by increasing H_2_S level in the hippocampus through giving H_2_S donor or inhaling H_2_S (32). However, whether plasma H_2_S levels are changed in patients with depression and its association with the severity of depression remains unknown. In this study, we further explored the role of H_2_S signaling in the pathophysiology of depression by investigating whether (1) plasma H_2_S level was altered in patients with depression and (2) there were any relationships between H_2_S levels and depressive symptoms in these patients.

## Method

### Subjects

Forty-seven inpatients with acute depressive episode (male/female = 20/27) were recruited from Jiangxi Mental Hospital. Two psychiatrists have confirmed the diagnosis of depression based on the Structured Clinical Interview for DSM-IV Axis I Disorders (SCID). The exclusion criteria included any other axis I or axis II DSM-IV diagnoses, including schizophrenia, bipolar disorder, substance abuse, anxiety disorder and so on. Fifty-one healthy controls (male/female = 28/23), matched with the patients by gender, age, education years, and body mass index (BMI), were recruited from the local community. Subject with a personal or family history of mental illness was excluded from control group. The exclusion criteria for all participants also included current pregnancy, autoimmune, allergic and neoplastic diseases, as well as other physical diseases that had occurred in the past 3 months, including hypertension, diabetes, heart or brain infarction.

The 17-item Hamilton Depression Rating Scale (HAMD-17) was used to evaluate depressive symptoms for all subjects (Supplementary Table 1) (33). The severity of depression was ranked on a HAMD-17 score: mild depression (8–17), moderate depression (18–24), and severe depression (>24) (34). To investigate whether antidepressants affected plasma H_2_S level, the depressed patients were divided into an antidepressant-treatment subgroup (*n* = 31) and an antidepressant-naive subgroup (*n* = 16). Subjects who were free of any antidepressant treatment for at least 1 month were defined as antidepressant-naive patients.

The research was approved by the Institutional Review Board at Jiangxi Mental Hospital and carried out in accordance with the Declaration of Helsinki. A written informed consent was provided from each subject, or his or her parents/guardians.

### Measurement of Plasma H_2_S

Whole blood from subjects who fasted overnight was collected into tubes with EDTA. After collection, samples were centrifuged at 3,000 rpm for 5 min at the temperature of 4°C and then the plasma was separated, aliquoted, and stored at−80°C until analysis.

The concentration of H_2_S in plasma was measured using a monobromobimane method coupled with reversed-phase high-performance liquid chromatography (RP-HPLC) (35). Free H_2_S in the plasma was analyzed by RP-HPLC after derivatization with excess monobromobimane (MBB) to form stable sulfide dibimane derivative. 30 μL of sample was pipetted and mixed with 70 μL of 100 mM Tris-HCl buffer (pH 9.5, 0.1 mM DTPA), followed by addition of 50 μL of 10 mM MBB. The reaction was terminated by adding 50 μL of 200 mM 5-sulfosalicylic acid at 30 mins later. After centrifugation, the supernatant was determined using an Agilent 1,220 HPLC system (Agilent Technologies, Santa Clara, CA, USA) and an Agilent ZORBAX Eclipse XDB-C18 column. The content of plasma H_2_S was calculated based on sulfidedibimane standard curves.

### Statistical Analysis

Data were presented as mean ± standard deviation (SD) and analyzed with the Statistical Product and Service Solutions (SPSS) 18.0 software. We compared categorical variables between patients and healthy controls using a chi-squared test. The continuous variables that were distributed normally were compared by Student's *t*-test and the independent variables that did not fit the normal distribution were analyzed by Kolmogorov-Smirnov and Mann-Whitney U tests. The relationships between plasma H_2_S and other variables were determined by Pearson correlation analysis and the independent relationships were analyzed by multivariate linear regression analysis. The level of significance was set at *P* < 0.05.

## Results

Forty-seven inpatients with depression (21 male, 26 female) and 51 healthy controls (28 male, 23 female) was enrolled in this study. Table 1 shows the demographic variables and the clinical values of control group and depressive group. There was no significant difference between two groups in terms of gender, age and BMI. The mean HAMD-17 score in depressive patients was statistically higher than that in the control group (22.15 ± 8.45 in depressive group *vs*. 3.22 ± 2.24 in control group, *P* < 0.001).

**Table 1:** Comparison of demographic and clinical variables in controls and patients.

**Variables**	**Control group**	**Depressive group**	**Statistic value**	***P* value**	**Effect size**
	**(*n* = 51)**	**(*n* = 47)**			
Gender (M/F)	28/23	21/26	Chi-squared test, *x^2^* = 1.022	0.312	–
Age (years)	38.02 ± 10.77	35.02 ± 13.98	*t*-test, *t* = 1.195	0.235	Cohen's *d* = 0.240
Education	11.01 ± 2.96	11.57 ± 3.57	U test, Z = −0.544	0.586	*r* = −0.085
Illness duration (years)	–	5.13 ± 3.66			
BMI (kg/m^2^)	21.30 ± 1.92	21.59 ± 1.96	*t*-test, *t* = −0.760	0.449	Cohen's *d* = −0.154
HAMD-17 score	3.22 ± 2.24	22.15 ± 8.45	*t*-test, *t* = −15.423	<0.001	Cohen's *d* = −3.059
H_2_S level (μmol/L)	1.02 ± 0.34	0.59 ± 0.29	*t*-test, *t* = 6.697	<0.001	Cohen's *d* = 1.359

The plasma level of H_2_S in the depressive patients was significantly lower than that in healthy controls (patients: 0.59 ± 0.29 μmol/L, controls: 1.02 ± 0.34 μmol/L; *t* = 6.697, *P* < 0.001) (Table 1). No significant difference was observed in plasma H_2_S level between male and female in both groups (both *P* > 0.05). For depressive patients, the level of plasma H_2_S was not different between antidepressant-treatment and antidepressant-naïve subgroup (*t* = 0.218, *P* = 0.828). A two-way ANOVA for H_2_S level in depressive patients showed that there was no significant main effect of gender (*F*_(1, 43)_ = 2.384, *P* = 0.130), no significant main effect of antidepressant treatment (*F*_(1, 43)_ = 0.036, *P* = 0.851) and no main effect of gender × antidepressant treatment (*F*_(1, 43)_ = 0.731, *P* = 0.397).

**Figure 1:**
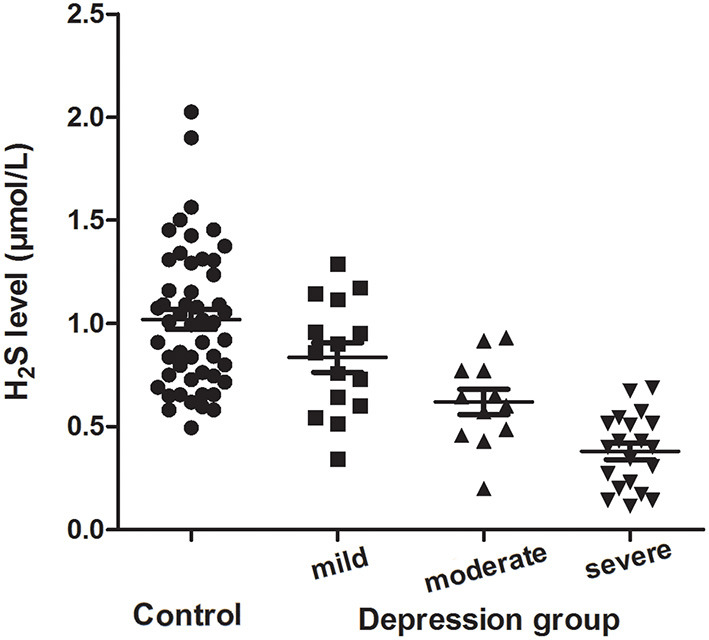
Plasma H_2_S levels (mean ± SD) in the controls and the mild, moderate and severe depression group.

Among 47 depressive patients, 15 patients (31.9%) had mild depression, 12 patients (25.5%) had moderate depression, and 20 patients (42.6%) had severe depression. The level of plasma H_2_S in mild, moderate and severe depressive patients was 0.84 ± 0.28, 0.62 ± 0.21 and 0.38 ± 0.18 μmol/L, respectively. One-way ANOVA revealed that there were significant differences among healthy controls, mild depressive, moderate depressive and severe depressive patients (*F*_(3, 97)_ = 24.984, *P* < 0.001). Bonferroni *post hoc* multiple tests for depressive subgroups showed that there was a significant decreased trend of the plasma H_2_S level among mild depressive patients compared to moderate depressive patients (*P* = 0.047), and moderate depressive patients compared to severe depressive patients (*P* = 0.015) (Figure 1).

**Figure 2:**
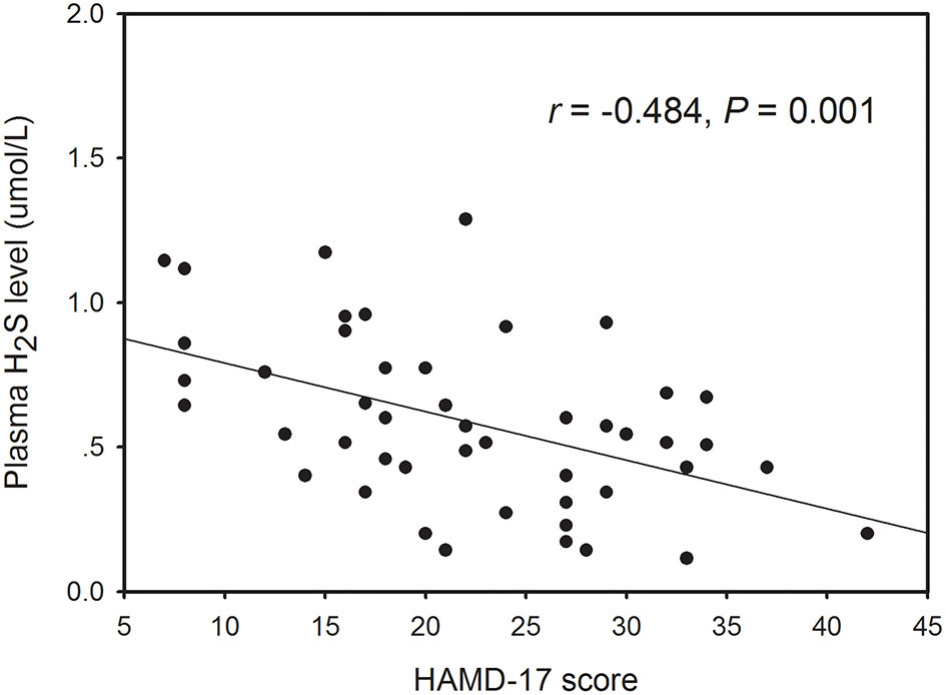
The correlation between plasma H_2_S levels and HAMD-17 scores in patients with depression.

Within the healthy control subjects, there no significant correlation between plasma H_2_S level and any demographic variable including gender, age, and BMI. However, Pearson correlation analysis revealed that the plasma H_2_S level was significantly correlated with age (*r* = −0.296, *P* = 0.043; Supplementary Figure 1) and HAMD-17 score (*r* = −0.484, *P* = 0.001; Figure 2) in patients with depression. Partial correlation analysis showed that the correlation between H_2_S levels and theHAMD-17 scores was still significant when controlling for gender, age, education years, BMI, and duration of illness (*r* = −0.374, *P* = 0.015). Finally, we conducted multivariate regression analysis to elucidate independent determinants of HAMD-17 scores (*R*^2^ = 0.586) and found that plasma H_2_S was an independent contributor to the HAMD-17 scores (*B* = −0.360, *t* = −2.550, *P* = 0.015) (Table 2).

**Table 2:** Correlations between plasma H_2_S levels, demographic characteristics and clinical variables in patients with depression.

**Variables**	**HAMD-17 score**		
	***B* (95% CI)**	***t***	***P* value**
Gender (M/F)	−0.135 (−0.399, 0.128)	−1.039	0.305
Age (years)	0.210 (−0.091, 0.512)	1.410	0.166
Education	−0.038 (−0.324, 0.247)	−0.271	0.788
Duration of illness (years)	−0.271 (−0.569, 0.027)	−1.841	0.073
BMI (kg/m^2^)	0.096 (−0.190, 0.383)	0.680	0.501
Plasma H_2_S	−0.360 (−0.646, −0.075)	−2.550	0.015

## Discussion

Previous studies have demonstrated that H_2_S is implicated in the pathophysiology of depression in rodents (30–32). In this study, the plasma levels of H_2_S were determined in Chinese patients with depression. We found a significant decrease in plasma H_2_S level in depressive patients compared to healthy controls, and decreased plasma H_2_S level was significantly correlated with the severity of depression.

H_2_S is an endogenous gasotransmitter with numerous homeostatic functions, such as neurotransmission and neuromodulation (24). A large number of studies have demonstrated that a dysfunction of H_2_S signaling takes a part in the pathophysiology of many neuropsychiatric disorders. The H_2_S level was decreased in the hippocampus of Alzheimer's disease (AD) mice and treating AD mice with NaHS reversed the impaired hippocampal synaptic plasticity and cognitive function (29, 36). Plasma H_2_S level is significantly decreased in both schizophrenia and AD patients, and has a correlation with the severity of cognitive impairments in these patients (35, 37). Hou et al. reported that endogenous H_2_S was decreased in the hippocampus of depressive model rats and responsible for the depressive-like behaviors of rats (32). In consistent with these results, we here showed that plasma H_2_S levels were significantly decreased in depressed patients and were correlated with the severity of depressive symptoms of patients, providing evidence for the contribution of H_2_S signaling to the pathogenesis of depression. It should be noted that change of plasma H_2_S in patients might also result from the treatment of antidepressants. However, we enrolled inpatients with acute depressive episode who had HAMD scores >8 in this study. Although some of the patients were taking antidepressants at the time of inclusion, the HAMD score showed that they were still depressed, suggesting that current antidepressants they used were not effective in improving their depressive symptoms. Indeed, meta-analyses of clinical trials have reported that more than 60% of patients fail to obtain significant or sustained remission with any single traditional antidepressant drug, with approximately one third of all depressed individuals failing two or more first-line antidepressant courses of treatment, consistent with the diagnosis of treatment-resistant depression (TRD) (38, 39). Our present study found that the level of plasma H_2_S was not different between antidepressant-treatment and antidepressant-naive subgroups in depressive patients, indicating that antidepressants alone do not affect plasma H_2_S levels in those patients whose depressive symptoms have not improved significantly. Therefore, in combination with the finding that endogenous H_2_S was decreased in the hippocampus of depressive rats (32), we postulate that change of plasma H_2_S level in patients is related to the illness *per se*, rather than secondary to antidepressant treatment. However, the mechanisms underlying the reduction of H_2_S in depression are still needed further investigations.

The HAMD is the most widely used scale for patient selection and follow-up in depression treatment studies (40, 41). We used HAMD-17 to evaluate the severity of depressive symptoms in the present study. Correlation analysis showed that there was a significantly negative correlation between plasma H_2_S levels and the HAMD-17 scores in depressive patients. Partial correlation analysis demonstrated that the correlation between H_2_S levels and the HAMD-17 scores was still significant when controlling for gender, age, education years, BMI, and duration of illness. Multivariate linear regression analysis revealed that plasma H_2_S level was negatively associated with HAMD-17 score. These results suggest that patients with lower H_2_S levels would be more likely to have severer depressive symptoms. Furthermore, the level of plasma H_2_S was decreased gradually from mild depression to moderate depression, and from moderate depression to severe depression, also indicating that plasma H_2_S is associated with the severity of depression. Therefore, the plasma H_2_S level may be served as a biomarker to evaluate the severity of depression.

There are some limitations in this study. First, the sample size was relatively small and all subjects were recruited from a single hospital. Replication in larger and multicenter samples is required to validate this conclusion. Second, H_2_S levels were measured in plasma, but not in the brain. Whether H_2_S level in the brain changes parallel with the level in plasma in patients is still unclear. Third, this was across-sectional study. Future studies are needed to elucidate the role of plasma H_2_S in the progression of depression. Additionally, although an association of decreased plasma H_2_S and the severity of depressive symptoms in patients with depression was found in this study, the mechanisms through which H_2_S affects depressive behaviors are needed to be investigated.

## Conclusion

Our present study shows that patients with depression have lower plasma H_2_S levels than healthy controls, and decreased H_2_S was associated with the severity of depressive symptoms inpatients. These results demonstrate an important role of H_2_S signaling in the pathophysiology of depression, suggesting that plasma H_2_S level may be a potential biomarker for the severity of depression.

## Data Availability Statement

The raw data supporting the conclusions of this article will be made available by the authors, without undue reservation.

## Ethics Statement

The studies involving human participants were reviewed and approved by the Institutional Review Board at Jiangxi Mental Hospital. Written informed consent to participate in this study was provided by the participants' legal guardian/next of kin.

## Author Contributions

Y-JY, C-NC, J-QZ, Q-SL, YL, and S-ZJ participated in clinical data collection and lab data analysis. Y-JY and BW designed the study, analyzed the data, and prepared the manuscript. All authors have read and approved the final manuscript.

## Funding

This research was supported by grants from the National Natural Science Foundation of China (82060258 and 81760254). It was also supported by the Jiangxi Provincial Natural Science Foundation (20202BAB206026, 20202BAB216012, 20202BBG73022, and 2020BCG74002), the Fujian Provincial Natural Science Foundation (2019J01164) and the Scientific Foundation of Quanzhou City for High Level Talents (2019C075R).

## Conflict of Interest

The authors declare that the research was conducted in the absence of any commercial or financial relationships that could be construed as a potential conflict of interest.

## Publisher's Note

All claims expressed in this article are solely those of the authors and do not necessarily represent those of their affiliated organizations, or those of the publisher, the editors and the reviewers. Any product that may be evaluated in this article, or claim that may be made by its manufacturer, is not guaranteed or endorsed by the publisher.
