# Supplementary material for: Decreased Plasma Hydrogen Sulfide Level Is Associated With the Severity of Depression in Patients With Depressive Disorder
Source: Front Psychiatry. 2021 Nov 11;12:765664. doi: 10.3389/fpsyt.2021.765664 (PMC8631961; doi:10.3389/fpsyt.2021.765664)
Supplement: Supplementary file 1 [file Presentation_1.PDF]

Decreased plasma hydrogen sulfide level is associated with the severity of depression in patients with depressive disorder

Supplementary materials

Supplementary Table 1 Hamilton Rating Scale for Depression (HAMD-17)

Item No.	Symptom	Score Range	Score
1	Depressed mood	0-4	
2	Feelings of guilt	0-4	
3	Suicide	0-4	
4	Early insomnia	0-2	
5	Middle insomnia	0-2	
6	Late insomnia	0-2	
7	Work and activities	0-4	
8	Retardation	0-4	
9	Agitation	0-4	
10	Psychicanxiety	0-4	
11	Somatic anxiety	0-4	
12	Gastrointestinal somatic symptoms	0-2	
13	General somatic symptoms	0-2	
14	Genital symptoms	0-2	
15	Hypochondriasis	0-4	
16	Loss of weight	0-2	
17	Insight	0-2	
Total HRSD score			

Grading		
0-4	0	Absent
	1	Mild or trivial
	2	Moderate
	3	
	4	Severe
0-2	0	Absent
	1	Slight or doubtful
	2	Clearly present

Reference:

Hamilton, M. (1960). A rating scale for depression. *J Neurol Neurosurg Psychiatry* 23**,** 56-62.

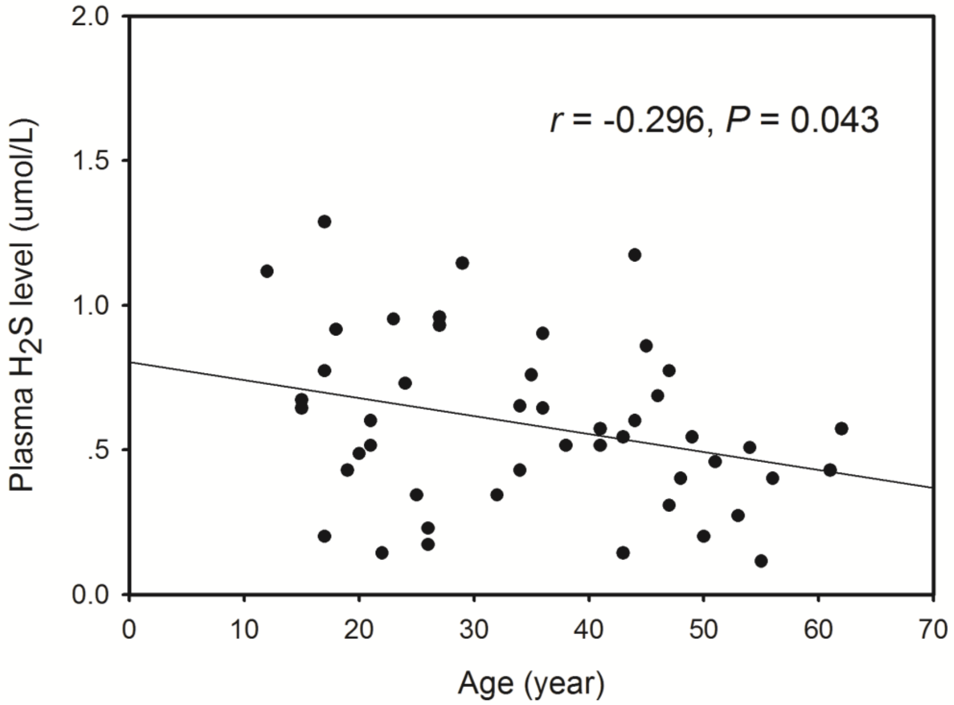

Supplementary Figure 1 The correlation between plasma H_2S level and age in patients with depression.
